# Rewiring cell-free metabolic flux in *E. coli* lysates using a block—push—pull approach

**DOI:** 10.1093/synbio/ysad007

**Published:** 2023-04-17

**Authors:** Jaime Lorenzo N Dinglasan, Mitchel J Doktycz

**Affiliations:** Biosciences Division, Oak Ridge National Laboratory, Oak Ridge, TN, USA; Graduate School of Genome Science and Technology, University of Tennessee-Knoxville, Knoxville, TN, USA; Biosciences Division, Oak Ridge National Laboratory, Oak Ridge, TN, USA

**Keywords:** lysate-based, cell-free metabolic engineering, maximum yield, biotransformation, block—push—pull

## Abstract

Cell-free systems can expedite the design and implementation of biomanufacturing processes by bypassing troublesome requirements associated with the use of live cells. In particular, the lack of survival objectives and the open nature of cell-free reactions afford engineering approaches that allow purposeful direction of metabolic flux. The use of lysate-based systems to produce desired small molecules can result in competitive titers and productivities when compared to their cell-based counterparts. However, pathway crosstalk within endogenous lysate metabolism can compromise conversion yields by diverting carbon flow away from desired products. Here, the ‘block—push—pull’ concept of conventional cell-based metabolic engineering was adapted to develop a cell-free approach that efficiently directs carbon flow in lysates from glucose and toward endogenous ethanol synthesis. The approach is readily adaptable, is relatively rapid and allows for the manipulation of central metabolism in cell extracts. In implementing this approach, a block strategy is first optimized, enabling selective enzyme removal from the lysate to the point of eliminating by-product-forming activity while channeling flux through the target pathway. This is complemented with cell-free metabolic engineering methods that manipulate the lysate proteome and reaction environment to push through bottlenecks and pull flux toward ethanol. The approach incorporating these block, push and pull strategies maximized the glucose-to-ethanol conversion in an *Escherichia coli* lysate that initially had low ethanologenic potential. A 10-fold improvement in the percent yield is demonstrated. To our knowledge, this is the first report of successfully rewiring lysate carbon flux without source strain optimization and completely transforming the consumed input substrate to a desired output product in a lysate-based, cell-free system.

## Graphical Abstract

**Figure:**
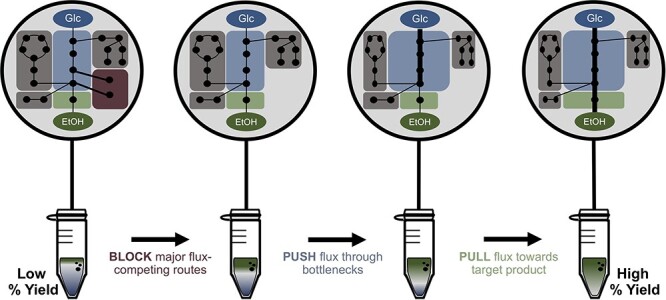

## 1. Introduction

Cell-based manufacturing systems routinely produce fuels and pharmaceutical precursors and products, among other commercially valuable compounds, from readily available substrates (1–3). These biomanufacturing systems are commonly evaluated by their productivity (the rate of product formation per unit volume), titer (concentration) and yield (the amount of product formed per substrate amount consumed) (1–3). However, engineering a living organism’s complex dynamic metabolism while meeting its survival demands typically requires multiple time-consuming design–build–test–learn (DBTL) cycles to make modest gains in these performance metrics (4, 5). For example, ethanol is a major biofuel, and realizing production at high titers (>40 g/l), productivities (>1 g/l/h) and yields (>90% of the maximum theoretical amount of ethanol that can be formed from the substrate) in cell-based systems has taken decades of strain optimization (6, 7). Cell-free optimizations and implementations show promise in expediting the generation of biosynthetic platforms with high production metrics (6, 8).

Cell-free or *in vitro* systems employ the components necessary to carry out metabolism outside of a living cell or in a ‘cell-free’ space (4, 9, 10). These systems are commonly generated either as reconstituted pathways from purified enzymes (i.e. reconstituted enzyme systems) or as crude cell extracts (i.e. lysate-based systems) via lysis procedures that preserve the functionalities of select cellular components (4, 5). In either case, cell-free systems are open biochemical environments devoid of organismal survival demands. Their amenability to direct manipulation offers unique and straightforward metabolic engineering approaches, and hence they are considered to have high potential for commercially viable production of diverse materials (6). For example, lysates have been used to synthesize several commodity chemicals such as 2,3-butanediol and styrene at high titers and productivities (11, 12). Additionally, cell-free systems can be lyophilized and stored at room temperature, which would eliminate the need for a cold chain and enable distributed biomanufacturing (13). This is empowered by ongoing efforts to improve the shelf-stability of these platforms for on-demand small-molecule synthesis, especially in times of supply chain disruption (14).

Implementing cell-free systems requires overcoming key barriers. When rebuilding large conversion pathways, reconstituted enzyme systems can be constrained by high enzyme production and purification costs, as well as problems associated with cofactor turnover (6, 11). On the other hand, lysates typically contain endogenous central metabolic pathways and cofactor regeneration reactions that feed into reactions catalyzed by supplemented heterologous enzymes. Consequently, protein purification requirements are greatly reduced albeit at the expense of reaction crosstalk. However, a significant barrier to production in lysate-based systems is the competition for flux between unwanted endogenous background reactions and target production modules, which inevitably impacts their yield (15). Making high-yield targeted conversions would require insulating an endogenous pathway by removing only a few enzymes that redirect flux through undesired reactions (6, 15). The ability to do so could benefit the rapid cell-free synthesis of high-value metabolites, which commonly have precursors in central metabolism, without arduous whole pathway purification (16, 17). To advance the commercial potential of extracts for large-scale or distributed biomanufacturing, methods to eliminate unwanted pathway crosstalk and rewire flux through target endogenous metabolic pathways in lysates are needed.

‘Block—push—pull’ approaches are commonly applied when engineering cellular metabolism for insulating target pathways and maximizing conversion yields (18–21). In this convention, to ‘block’ means to inactivate reactions by removing endogenously expressed enzymes (commonly by gene deletions) that interface with target and flux-competing pathways. To ‘push’ means to drive flux forward by overexpressing flux-limiting enzymes, and to ‘pull’ is to direct flux toward the end-product by overexpressing enzyme(s) in a terminal pathway module (20, 21). These cell-based metabolic engineering strategies however take a toll on host vitality and require laborious molecular cloning procedures, lengthening and increasing DBTL cycles (4). The open nature of lysate-based cell-free systems offers unique push and pull strategies to redirect flux without imposing metabolic burden on source strains. For example, bottlenecks can be overcome, and specific pathway modules can be enriched by tailoring the reaction environment (i.e. cofactor availability and pH) to enhance enzyme activity, manipulating source strain cultivation conditions and supplementing lysates with overexpressed proteins (17, 22–24). On the other hand, reaction blocking strategies that effectively shave endogenous lysate metabolic complexity while taking advantage of the manipulable cell-free environment are lacking. Prior cell-free metabolic engineering (CFME) efforts that focused on rewiring carbon flux typically involved deriving lysates from ‘knock-out’ strains (15, 25). However, this strategy will not always result in predicted changes to lysate metabolism presumably because gene deletions lead to protein expression changes that rearrange flux distributions and metabolic networks in the source strain (26). This method is also constrained by cell viability and thus faces limitations common to cell-based metabolic engineering (15). Recently, negative clustered regularly interspaced short palindromic repeats effectors that downregulate by-product-forming enzymes in source strains were shown to rewire metabolic flux in a cell-free system toward target end-products without affecting source strain growth (27). While this improved product titers, the substrate-to-product conversion yields remained low because this strategy can only ‘knock-down’, and not completely eliminate, flux-competing reactions (27). Inhibiting enzymes in cell-free reactions may be a viable strategy for inactivating unwanted reactions without impacting source strain phenotypes. This approach also benefits from being able to add inhibitors directly into the open lysate environment. However, the cost and availability of these molecules could be restrictive (28). Another recently described strategy for reducing concentrations of select enzymes in extracts involves the incorporation of an affinity tag at the N- or C-terminus of endogenously expressed enzymes for subsequent removal from the lysate (29). Importantly, genomic integration of the 6xHis-tags had no significant effect on source strain viability, even though central metabolic, pyruvate-consuming enzymes were targeted. When the derived lysates were fed glucose, the formation of unwanted by-products was slowed, enabling significant pyruvate accumulation for a few hours followed by eventual by-product pooling. This affinity-based selective enzyme depletion method can theoretically be improved to completely remove these unwanted reactions post-lysis, but this has yet to be reported.

**Figure 1.**
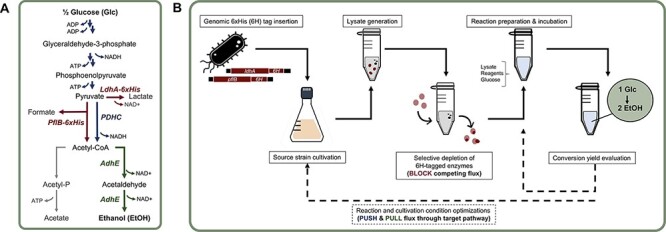
(**A**) The simplified metabolic pathway for endogenous ethanol biosynthesis from glucose in *E. coli* lysates. The strain used here was genome-engineered to express endogenous LdhA and PflB proteins with terminal 6xHis-tags for selective removal from the derived lysate (i.e., blocking). Arrows illustrating the conversion of glucose to acetyl-CoA represent reactions that flux will be pushed through. Flux will be pulled from acetyl-CoA to ethanol through the AdhE-catalyzed reactions. (**B**) The schematic of the cell-free block—push—pull approach employed here to maximize the ethanol yield from glucose.

Here, we describe a cell-free block—push—pull approach that insulates and drives flux through an endogenous lysate metabolic pathway for high-yield metabolic conversion. This is demonstrated with ethanol synthesis from glucose using an *Escherichia coli–*derived extract. To achieve this, the affinity-based selective enzyme depletion strategy was optimized to block unwanted reactions, those that compete with flux toward ethanol synthesis. This optimization was performed in lysates derived from a previously described *E. coli* strain that encodes an N-terminal 6xHis-tag at its pyruvate formate lyase B (*pflB*) and lactate dehydrogenase A (*ldhA*) genes. The reaction blocking strategy was then complemented with existing CFME strategies (i.e. reaction and source strain cultivation condition optimizations) that drive flux through bottlenecks and toward ethanol without further genetic manipulations (Figure 1). Removing unwanted reactions and modifying reaction or cultivation parameters to focus and increase flux through the pathway successfully modified a low ethanol-producing *E. coli* lysate into a homoethanologenic cell-free system that produces ethanol from glucose with >90% yield.

## 2. Materials and methods

### 2.1 Cell culture

The strain, derived from *E. coli* BL21 (Star) DE3, used here was previously engineered via a pORTMAGE protocol to incorporate genomic 6xHis-tag DNA sequence insertions upstream of the *ldhA* and *pflB* genes (29). These cells were initially grown in standard 2xYPT-G media (16 g/l tryptone, 10 g/l yeast extract, 5 g/l NaCl, 7 g/l KH_2_PO_4_, 3 g/l K_2_HPO_4_ and 18 g/l glucose). Glucose concentrations in the media were adjusted to 9 g/l or 27 g/l according to our source strain cultivation condition optimization protocol. Starter cultures in 6 ml 2xYPTG were incubated overnight at 30°C with shaking at 250 rpm. Baffled Erlenmeyer flasks containing 100 ml of the same media were inoculated with 2% of the overnight culture and incubated at 37°C with constant shaking at 250 rpm. Cell growth was monitored by measuring the optical density of cultures at 600 nm (OD_600_). Cells were pelleted by centrifugation for 5 min at 10 000 g at 4°C, initially between OD_600_ 5.8 and 6.0. When optimizing this parameter, cells were strictly grown to either OD_600_ 5.0 or 7.0 and then between OD_600_ 5.0 and 5.2 for subsequent experiments. Cell pellets were washed twice with cold S30 buffer without dithiothreitol (DTT) (14 mM magnesium acetate, 60 mM potassium acetate and 10 mM Tris-acetate; pH 8.2) and then weighed. These were flash-frozen and stored at −80°C unless lysed on the same day. Fresh cultures were prepared for each optimization step.

### 2.2 Lysate preparation

Cell pellets were resuspended in 0.8 ml S30 buffer (without DTT) per gram wet cell mass, after thawing on ice. Resuspensions were sonicated using a Branson 450 sonifier with a 3.2-mm tip. Briefly, 1 ml resuspension was placed in 1.5-ml microcentrifuge tubes surrounded by an ice water bath. Cells were lysed by applying 590 J at 50% tip amplitude at 10-s ON and 10-s OFF intervals directly to the resuspension. The resulting extracts were centrifuged twice at 21 100 g for 10 min at 4°C. The supernatant was aliquoted to clean microcentrifuge tubes, flash-frozen and then stored at −80°C until use. Extracts were prepared from fresh cultures for each optimization step.

### 2.3 Lysate depletion

For optimizing the post-lysis depletion method, HisPur™ Cobalt Resin (Thermo Fisher Scientific) suspensions were washed with 600 µl S30 buffer (without DTT) on ice and centrifuged for 30 s at 21 100 g at 4°C. It should be noted that the resin is suspended in ethanol in storage. In initial experiments, all resin suspension volumes were washed twice following our previously reported protocol, but this was insufficient for higher-volume resin suspensions as residual ethanol storage buffer affected our time-zero sample measurements (29) (Figure S1). More wash cycles were performed to remedy this problem (Table S1). The number of wash steps was thus optimized per resin suspension volume to ensure the removal of ethanol buffer used for resin storage. LdhA–6xHis and PflB–6xHis were depleted from lysate proteomes by adding 1X volume of the cell extract to Co^2+^ beads washed from 0.2X, 0.6X, 1X, 1.4X and 1.8X volume of ice-cold resin suspension. A fixed lysate volume of 200 µl was used. Lysates were initially mixed with beads for 1 h at 4°C with shaking at 900 rpm. The incubation time was changed to 30 min after the optimization of this protocol for subsequent experiments. All lysate–bead mixtures were centrifuged at 21 100 g for 30 s. Supernatants were aliquoted, flash-frozen and stored at −80°C until use. The depletion method was performed in triplicate on the same batch of extract. Replicates are referred to here as ‘depletion replicates’.

### 2.4 CFME reaction preparation

All cell-free metabolic reactions were prepared with 18 mM magnesium glutamate, 15 mM ammonium glutamate, 195 mM potassium glutamate, 150 mM Bis-Tris and 10 mM dipotassium phosphate. Lysate was added to these mixtures at a final concentration of 4.5 mg/ml unless otherwise noted. For every tested condition, reactions were prepared with depletion replicate lysates (*n* = 3) to evaluate variability in the depletion strategy. Cofactor [nicotinamide adenine dinucleotide (NAD^+^), coenzyme A (CoA) and adenosine triphosphate (ATP)] and glucose concentrations and pH levels used to initiate the reactions are detailed in the manuscript. To achieve acidic pH levels, glacial acetic acid was added to S30 buffer solutions that were then used to prepare the reactions. The pH of the reaction itself was measured using Hydrion MicroFine pH papers prior to incubation. Reactions were carried out in 30 µl volume in enclosed 1.5-ml microcentrifuge tubes (Thermo Scientific; #3451), 0.2-ml polymerase chain reaction (PCR) tubes (VWR; #20170-012) or 50 µl wells on a 384-well plate (Corning; #3544) sealed with polyester film (VWR #75853-868) as described. Reactions were incubated at 37°C for 20 h unless otherwise stated. For each replicate, a control time-zero reaction was prepared as described previously (30). Briefly, lysate metabolic activity was terminated with 5% trichloroacetic acid (TCA) prior to the addition of glucose to prepare controls.

### 2.5 Metabolite analyses

CFME reactions were quenched with an equivalent volume of 5% TCA and then diluted with an equal volume of ultrapure water. High-performance liquid chromatography (HPLC) was used to measure changes in glucose, ethanol, lactate and acetate concentrations between time-zero and end-point reactions. Measurements were taken using an Agilent 1260 Infinity system (Agilent, Santa Clara, CA) equipped with an Aminex HPX 87H column (Bio-Rad, Hercules, CA) for carbohydrate, organic acid and alcohol analyses. An isocratic method using 5 mM sulfuric acid was used to elute target metabolites from the column at a flow rate of 0.55 ml/min for 30 min at 35°C. Metabolites were detected using a refractive index detector (Agilent, Santa Clara, CA). Peak areas were extracted using manual integration for glucose peaks and automatic integration for lactate, acetate and ethanol peaks in the HPLC system’s companion software. Concentrations were calculated using standard calibration. Reported values for metabolite consumption and production are changes in concentrations between end-point and time-zero reactions. Student’s *t*-tests were used to evaluate statistical significance (*P* < 0.05). The percent ethanol yield from glucose was calculated as follows, given that 1 mol glucose can theoretically be converted to 2 mol ethanol in *E. coli* lysates:


(1) $$\% \,Yield_{\left({\frac{EtOH}{Glc}}\right)} = \frac{{Actual\ yield_{\left(\frac{EtOH}{Glc}\right)}}}{Theoretical\ yield_{\left(\frac{EtOH}{Glc}\right)}} \times 100$$

(2) $$Actual\ yield_{\left(\frac{EtOH}{Glc}\right)} = \frac{mol\ EtOH\ produced}{mol\ Glc\ consumed}$$

(3) $$Theoretical\ yield_{\left(\frac{EtOH}{Glc}\right)} = \frac{2\ mol\ EtOH}{1\ mol\ Glc}$$

## 3. Results and discussion

### 3.1 Blocking flux-competing pathways via selective depletion of LdhA and PflB proteins in lysates redirects carbon flow to ethanol

Glucose-derived carbon flow could theoretically be diverted toward ethanol by removing PflB and LdhA and associated flux toward formate and lactate. However, the ratio of NAD+ to its reduced equivalent, nicotinamide adenine dinucleotide hydrogen (NADH), or [NAD^+^]-to-[NADH] ratio, must be balanced to sustain flux toward ethanol. Conventionally, for 1 mol glucose consumed, glycolysis generates 2 mol of NADH that is primarily recycled to NAD^+^ via the lactate-forming LdhA enzyme (Figure 1A). Because this reaction sustains glycolysis by providing NAD^+^, lactate has been observed as a major fermentation product in the lysate metabolism of glucose (30). To redirect flux toward ethanol, an alternative ethanol-forming, [NAD^+^]/[NADH] balancing pathway can be activated through the pyruvate dehydrogenase complex and aldehyde-alcohol dehydrogenase (PDHC–AdhE) pathway module (29, 31). Briefly, PDHC generates 2 mol acetyl-CoA and 2 mol NADH from 2 mol pyruvate (Figure 1A). The net output of glycolysis and PDHC is thus 4 mol NADH per mol glucose. AdhE can recycle the equivalent amount of NAD^+^ from 4 mol NADH when transforming 2 mol acetyl-CoA to 2 mol ethanol, sustaining glycolytic flux (31). A prerequisite for PDHC–AdhE activation is the inactivation of the PflB reaction. Although PflB is oxygen-sensitive, its activity in lysates and aerobic environments has been reported (29, 32, 33). Active PflB converts 1 mol pyruvate and 1 mol CoA to 1 mol acetyl-CoA, forming 1 mol formate in the process. Because this reaction does not generate NADH, subsequent ethanol formation via NADH-dependent AdhE is imbalanced with glycolysis (Figure 1A) (29, 31, 34).

**Figure 2.**
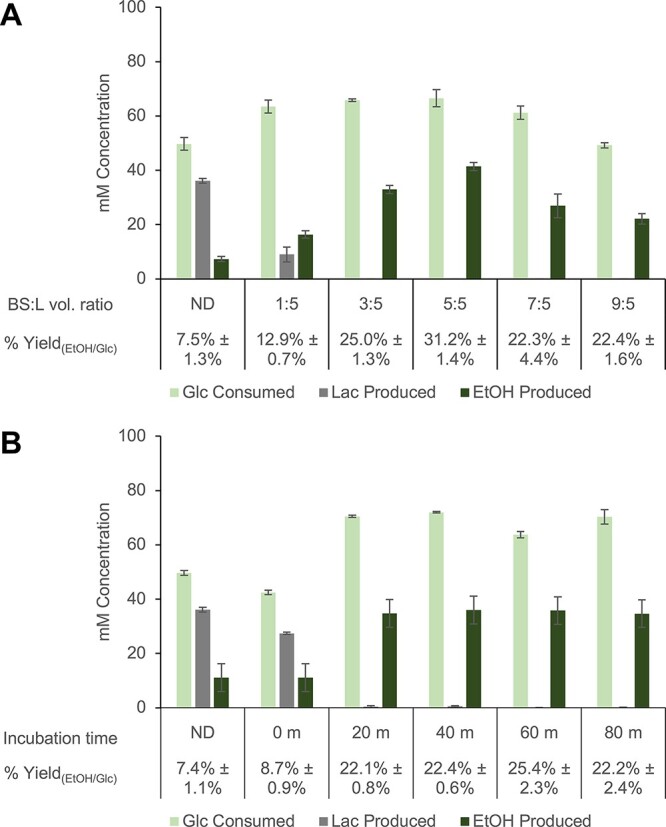
Optimization of the post-lysis lysate depletion method to selectively remove 6xHis-tagged PflB and LdhA and redirect lysate metabolic flux to ethanol. Normalized metabolite concentrations and calculated % ethanol yields in reactions made with (**A**) lysates that were exposed to varying amounts of Co^2+^ beads for 1 h and (**B**) lysates depleted with Co^2+^ beads washed from an equivalent suspension volume for different incubation times. All reactions were set up using 1 mM of NAD^+^, CoA and ATP and 100 mM glucose. Reactions were incubated at 37°C for 20 h. Replicates of the depletion method (i.e. ‘depletion replicates’) were used to prepare triplicate reactions for all conditions, and error bars represent standard errors. BS:L vol. ratio = bead suspension–to–lysate volume ratio; ND = non-depleted control.

The post-lysis affinity-based enzyme depletion method was optimized to remove 6xHis-tagged PflB and LdhA proteins from derived lysates to the point of inactivating these reactions, ensuring flux redistribution toward the PDHC–AdhE route of ethanol formation. In prior work, lysates were incubated with beads washed from a volume of HisPur Cobalt Resin suspension that was one-fifth of the lysate volume (29). This method however still resulted in some formate production and significant lactate accumulation after 24 h. Utilizing more Co^2+^ beads to bind 6xHis-tagged proteins in the lysate should help eliminate flux-competing reactions. To this end, Co^2+^ beads washed from varying volumes of resin suspension were mixed with a fixed volume of lysate derived from the genome-engineered strain, and mixtures were incubated for 1 h at 4°C under shaking conditions. The resulting supernatants (i.e. depleted lysates) were normalized to the same protein concentration and used to prepare CFME reactions that were fed with a glucose substrate. All metabolite concentrations quantified in incubated reactions were normalized against time-zero controls, which were prepared as described previously (30). Furthermore, the variability of the depletion approach was assessed by performing this method in triplicate from the same batch of lysate and then using the resulting ‘depletion replicates’ to prepare replicate cell-free reactions (Figure 2). Despite batch variability being an issue in other lysate-based applications (e.g. protein expression) (35), metabolic performances of extracts prepared from biological replicates (i.e. different cell cultures) do not significantly vary (29, 36).

Contrary to the previous report (29), no formate was detected in any of the modified extracts or in the unmodified extract. PflB activity in lysates has been described in the past, so it is likely that formate was only accumulated here at undetectable levels or was actively consumed by formate dehydrogenases (29, 30, 37). Since it has been previously shown that LdhA and PflB can be simultaneously purified from the lysate by this procedure, decreasing lactate buildup was used as a proxy for evaluating the purification of both proteins (29). Reactions prepared with lysates exposed to an equivalent volume of bead suspension resulted in no lactate accumulation and in the highest % ethanol yield from glucose (31.2% ± 1.4%). This is a significant 2-fold improvement (*P* < 0.01) compared to the % yield in the 1:5 volume ratio condition (12.9% ± 0.7%), which was the only ratio tested in the previous study (29) (Figure 2A). This condition optimally enabled the removal of by-product-forming activity while increasing flux through the PDHC route of acetyl-CoA formation. Notably, exposure to even more beads (7:5 and 9:5 volume ratios) lowered glucose consumption, ethanol production and % ethanol yield relative to the 5:5 condition (Figure 2A). This is possibly because of increased binding of background proteins in the lysate to the Co^2+^ beads (29). Nonetheless, all lysates processed using the 6xHis-tag purification strategy consumed more glucose and produced more ethanol when compared to a non-depleted (ND) lysate (Figure 2A). The effect of removing lactate-forming activity on the target yield increase is obvious in these data. To confirm the contribution of PflB depletion, the 5:5 volume ratio condition was also applied to lysates derived from a strain that endogenously expresses 6xHis-tagged LdhA only. Evidently, depleting both PflB and LdhA has a more positive impact on ethanol yield compared to LdhA depletion alone (Figure S2).

To determine whether increasing the exposure period of lysates to beads would improve selective enzyme depletion, lysates were mixed for varying durations with beads washed from an equivalent HisPur Co^2+^ resin suspension and then used to prepare CFME reactions (Figure 2B). No significant improvement in % yield beyond 20 min of incubation was observed (Figure 2B). The results also suggest that potential shear forces from shaking the beads vigorously in lysates have no effect on glucose metabolism or % ethanol yield; otherwise lysate performance could be expected to decrease with time (Figure 2B). Moving forward, a standard 30-min incubation period of lysates with beads from an equivalent volume of Co^2+^ resin suspension was used to prepare modified lysates. Extracts depleted under these conditions are henceforth referred to as ‘blocked’ lysates.

The reaction blocking strategy described here has several advantages over other tactics to rewire lysate metabolic flux. Gene knock-outs can have unpredictable effects on harvested lysate metabolism presumably because the source strain reconstructs its proteome to accommodate the deletion(s) (15, 25, 38, 39). These mutant lines would result in lysate proteomes that differ on a global scale when compared to the wild-type (39). Intuitively, tractable changes to the ‘static’ lysate proteome can be made by selectively removing enzymes post-lysis, enabling engineering designs with predictable outcomes and significantly simplifying the integration of lysate metabolic models. Displaying 6xHis-tags on endogenously expressed enzymes does not impact cell growth, whereas the number of gene knock-outs that can be introduced without strain optimization is limited (15). Additionally, simply reducing the concentrations of flux-competing enzymes by downregulating gene expression does not eliminate unwanted reactions in lysates (27, 29). The strategy presented here already provides >3-fold improvements in both titer and % yield compared to the unmodified extract even without the optimization of the reaction environment or heterologous enzyme expression, both of which were necessary for achieving a similar improvement in butanediol titer via a knock-down strategy (27).

### 3.2 Optimized reaction and cultivation conditions push and pull flux through the insulated pathway and improve ethanol yield in blocked lysates

Removing enzymes alone can be insufficient for achieving high conversion yields. Given the complexity of lysate metabolism, glucose-derived carbon can be trapped in metabolic intermediates or incorporated into less obvious end-products of central metabolism (30). Push and pull metabolic engineering strategies are typically implemented to improve pathway yields by overcoming bottleneck reactions while increasing carbon flow toward a target metabolite (18). Previous CFME efforts have shown that optimal reaction and source strain cultivation conditions can enrich the concentrations or activities of desirable enzymes in lysate-based systems, increasing flux through a pathway of interest (17, 22–25, 27). Some of these tactics were thus applied sequentially to increase flux through the insulated ethanol biosynthesis pathway in the blocked lysate, using % yield of ethanol from glucose as a parameter for optimization.

**Figure 3.**
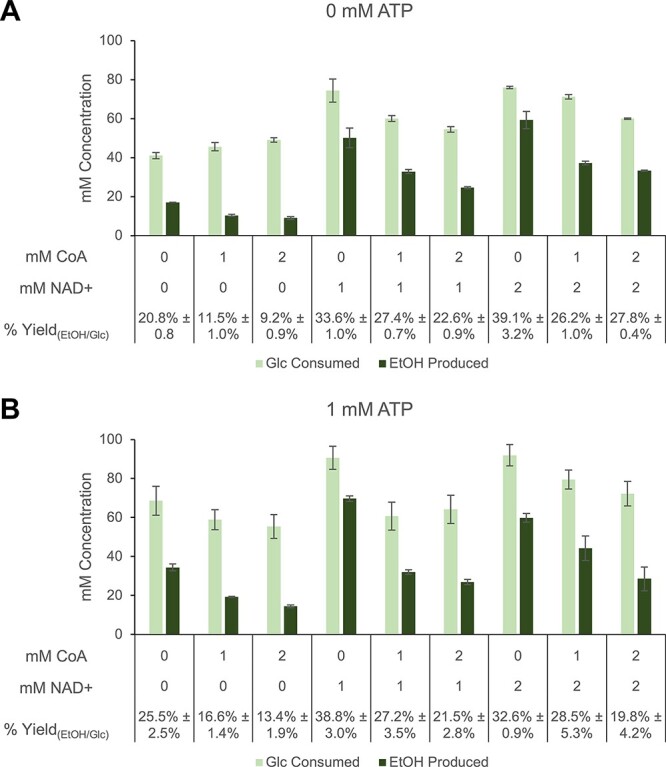
Optimization of cofactor concentrations in reactions prepared with blocked lysate to drive carbon flux toward ethanol. Normalized metabolite concentrations and calculated % ethanol yields in reactions of blocked lysates prepared with 100 mM glucose, different combinations of 0, 1 or 2 mM of NAD^+^ and CoA, and (**A**) 0 mM or (**B**) 1 mM ATP. Reactions were incubated at 37°C for 20 h. Reactions for all conditions were prepared using depletion replicates, and error bars represent standard errors (*n* = 3).

First, different combinations of starting cofactor concentrations were sampled in reactions prepared with glucose-fed, blocked extracts. The cofactors NAD^+^, ATP and CoA are all responsible for driving past flux-restricting steps in the glycolysis module of the target pathway (Figure 1A), hence the initial supplementation of 1 mM of each of these reagents to the reactions. Interestingly, ∼40% (39.1% ± 3.2%) of the maximum theoretical ethanol yield can be achieved when the reactions are started with 2 mM NAD^+^ (Figure 3A); supplementation with ATP or CoA is unnecessary for improving the yield. These conditions result in a % yield that is significantly higher (*P* = 0.03) than that of the initial condition (27.2% ± 3.5%) (Figure 3B). NAD^+^ concentrations beyond 2 mM had no positive effect on ethanol yield (Figure S3). And ∼40% yield could also be realized if 1 mM ATP and 1 mM NAD^+^ were added to reactions without CoA, suggesting a complex interplay between ATP and NAD^+^ availability (Figure 3B). Regardless of starting ATP or NAD^+^ concentrations, decreasing CoA concentrations consistently improved % ethanol yields (Figure 3A and B). This can be attributed to the regulatory role of CoA-derived moieties, which can bottleneck flux through glycolysis to ethanol. When accumulated, succinyl-CoA and acetyl-CoA inhibit the pyruvate-forming enzymes pyruvate kinase and PDHC, respectively (34, 40, 41). These intermediates can become excessive when there is high CoA availability at the start of the reaction and result in lower glucose consumption and ethanol production, thereby lowering % ethanol yields (Figure 3A and B). Another possibility is that free CoA inhibits the forward reaction catalyzed by the acetaldehyde dehydrogenase component of AdhE, stalling ethanol synthesis (42).

**Figure 4.**
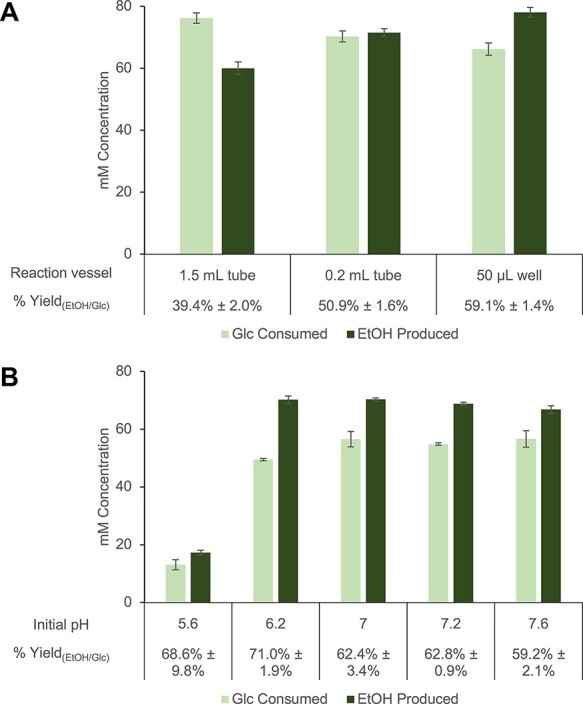
Optimization of the reaction vessels and initial reaction pH values to drive carbon flux toward ethanol in blocked lysates. Normalized metabolite concentrations and calculated % ethanol yields in reactions incubated in (**A**) different enclosed reaction vessels with varying airspace volumes and (**B**) sealed 50-µl wells with varying initial pH values. Reactions were supplemented with 2 mM NAD^+^ and 80 mM glucose and then incubated at 37°C for 20 h. Reactions for all conditions were prepared using depletion replicates, and error bars represent standard errors (*n* = 3).

Next, different reaction vessels were tested to determine whether smaller-sized reactors would benefit the system’s conversion efficiency. Decreasing the size of the vessel reduces the ratio of air/liquid surface area to reaction volume and the reaction headspace, both of which reportedly limit the oxygenation of cell-free reactions (43, 44). In *E. coli* cells, the electron transport chain and component oxygen-dependent enzymes that regenerate NAD^+^ from NADH are inactivated when oxygen is limited (34, 45–47). Increased [NADH]/[NAD^+^] in the cytosol is consequently regulated by the increased activity of endogenous AdhE enzymes (45, 47), a phenomenon that also likely occurs in oxygen-limited *E. coli* lysates (48). Additionally, a smaller air/liquid surface area and reaction headspace could better prevent ethanol evaporation from the reaction volume. Hence, 30 µl glucose-fed reactions prepared with blocked lysates and optimized cofactor concentrations were incubated in reaction vessels of varying sizes: 1.5-ml microcentrifuge tubes, 0.2-ml PCR tubes and 50-µl non-binding surface wells in a 384-well plate sealed with film. The ratios of air/liquid surface area to reaction volume of the 30-µl reactions in these vessels were ∼0.65, 0.42, and 0.32 mm, respectively. Expectedly, 30-µl reactions in 50-µl wells had a significantly higher mean % yield (59.1% ± 1.4%) compared to the average ∼40% yield of reactions in 1.5-ml microcentrifuge tubes (*P* < 0.01) (Figure 4A).

Cell-free systems provide the advantage of directly altering the reaction environment’s pH, which can potentially improve the activity of specific enzymes and therefore pathway performance. Sampling different initial reaction pH values has therefore become a common CFME strategy for improving product titers (12, 23, 27, 49). This strategy was employed here specifically to improve the ethanol yield in the blocked extract. *Escherichia coli* cells regulate cytosolic pH within a range of 7.2–7.8, hence the use of pH 7.2 as the initial condition (50). However, reducing the pH from 7.2 to 6.2 resulted in a significant % yield increase (*P* < 0.01) to 71.0% ± 1.9% (Figure 4B). This is attributed to a modest increase in the ethanol concentration (*P* > 0.05) and a significant decrease in glucose consumption (*P* < 0.01) (Figure 4B). In *E. coli* cells, a reduction from cytosolic pH 7.0 to 6.0 leads to a 20% decrease in glycolytic flux (51). Although the pH optima of enzymes *in vitro* are generally lower than that observed *in vivo*, it is conceivable that cell-free glycolytic enzymes are already slightly impaired at pH 6.2, leading to the reduced usage of glucose at this pH (52). Intriguingly, however, the decrease in glucose consumption had no significant effect on the ethanol concentration compared to the initial condition (Figure 4B). This suggests that carbon flux from consumed glucose is better directed toward ethanol in lysates with a slightly more acidic reaction environment. Further reducing the reaction pH results in a similar ethanol yield (68.6% ± 9.8%) but compromises ethanol concentration (Figure 4B). For these reasons, pH 6.2 was considered the optimum for succeeding experiments.

**Figure 5.**
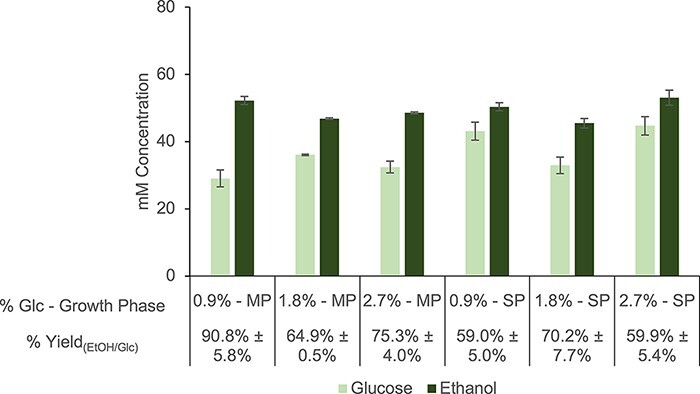
Optimization of source strain cultivation conditions to drive carbon flux toward ethanol in resulting lysate proteomes. Normalized metabolite concentrations and calculated % ethanol yields in blocked lysates derived from strains grown under different conditions. Reactions were prepared with optimized reaction conditions and 80 mM glucose and then incubated at 37°C for 20 h. Reactions for all conditions were prepared using depletion replicates, and error bars represent standard errors (*n* = 3).

To further improve ethanol yield, different source strain growth conditions were evaluated for enriching throughput of the target pathway. As reported, the lysate’s endogenous metabolic proteome can be curated by growing cells under different nutritional conditions or by harvesting cells at different growth stages, improving CFME outcomes (24, 36). Ethanologenic metabolism in *E. coli* cells is differentially expressed in cultures with varying concentrations of carbon substrate and growth phase (46, 53–55). Glucose-rich media stimulate glycolysis, but substrate-derived carbon is primarily funneled to lactate generation instead of ethanol synthesis under high glucose concentrations (53). *Escherichia coli* ethanol production is known to occur during fast growth on glucose even under aerobic conditions, but upregulated AdhE expression has also been documented in *E. coli* cultures at the onset of stationary phase (54, 55). The glucose concentration for growth and harvesting time could thus theoretically be optimized to obtain lysates with enriched ethanologenic activity. To this end, the strain with genomically integrated 6xHis-tags was grown in 2xYPT media containing different concentrations of glucose. Cells were then pelleted for lysis at mid-log growth (OD ∼ 5.0) and the onset of the stationary phase (OD ∼ 7.0). Blocked extracts were derived from these cells and were fed glucose in reactions using the optimized reaction conditions. A lysate with high ethanol yield from glucose (90.8% ± 5.8%) could be extracted from cells grown exponentially on 0.9% glucose (Figure 5). Compared to lysates that were initially harvested from mid-log cells grown on 1.8% glucose, the optimized lysate produced ethanol at significantly higher concentrations (*P* < 0.01), leading to the significantly higher % ethanol yield from glucose (*P* < 0.01) (Figure 5). This >90% theoretical yield is considered industrially relevant for ethanol biosynthesis (6, 7).

In efforts to maximize the redirection of glucose-derived carbon flux toward ethanol synthesis in crude lysates, reaction conditions defined as optimal should yield high glucose-to-ethanol transformation while minimizing acetate formation or consumption. In addition to ethanol production, changes in the system’s acetate concentrations were analyzed throughout optimization experiments. Both the production and the consumption of acetate in *E. coli* lysate–based cell-free systems have been reported (11, 25, 29, 30). Enzymes involved in the acetyl-CoA–acetate pathway can drive reactions in either direction depending on the lysate’s adenylate charge (30). That is, the pathway, which involves ATP and adenosine diphosphate exchange, can either form or deplete acetate depending on the availability of these adenosine cofactors (Figure 1A). When this pathway is driven forward, acetate is formed alongside ethanol since the two fermentative end-products share the same acetyl-CoA precursor. Consequently, acetate production is likely to compete with ethanol synthesis for glucose-derived carbon flux (56). When driven backward toward acetyl-CoA synthesis, acetate can be a secondary carbon source for ethanol formation since it is initially present in the reactions at a final concentration of ∼80 mM (25). This is because the S30 buffer used to prepare lysates consists of acetate salts. If acetate is indeed consumed in the reactions, this would obscure % ethanol yield from glucose calculations. When optimizing for starting cofactor concentrations, acetate was observed in the blocked extract. Like ethanol, acetate increased with decreasing CoA, reinforcing the assumption that glycolysis is bottlenecked by high CoA levels (Figure S4A). Incubating reactions in more oxygen-limited environments however increasingly drove the acetyl-CoA–acetate pathway module toward acetyl-CoA synthesis, as evidenced by acetate consumption in 50-µl vessels (Figure S4B). This could be explained by the need for an additional ATP consuming sink that is fulfilled by the acetate kinase reverse reaction when O_2_-dependent ATP hydrolases are inactivated in the system (30, 43). Despite acetate consumption in the sealed 50-µl wells, data from the initial reaction pH optimization experiment imply that carbon derived from this secondary source is likely not incorporated into ethanol since ethanol concentrations do not significantly change in conditions where acetate is produced (pH 6.2) or consumed (pH > 7.0) (Figure S4B and C). These data also reinforce the use of pH 6.2 for ethanol production in lysates since this condition has the least significant acetate production or consumption. Under these reaction conditions, acetate formation or consumption remained insignificant in extracts regardless of source strain cultivation conditions (Figure S4D).

To further ensure that ethanol in this optimized system was formed from glucose, reactions (*n *= 2) were incubated for 20 h without glucose and compared to respective time-zero controls. No glucose or ethanol was detected in any of the time-zero replicates, and only 1.9 ± 0.08 mM ethanol was formed after 20 h. This strongly suggests that ethanol is derived from glucose and not from carbon sources initially present in the reactions at high (>80 mM) concentrations (i.e. acetate and glutamate) (57).

### 3.3 Maximum glucose-to-ethanol conversion is consistent in the optimized system

**Figure 6.**
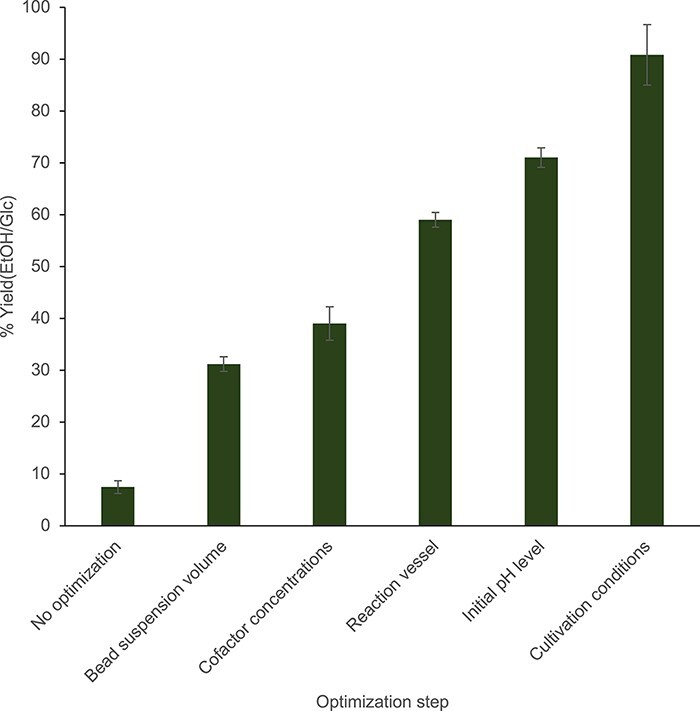
Summary of the additive improvements to % ethanol yields in the system due to conditions that optimally block, push and pull flux. Reactions for all conditions were prepared using depletion replicates, and error bars represent standard errors (*n* = 3).

**Figure 7.**
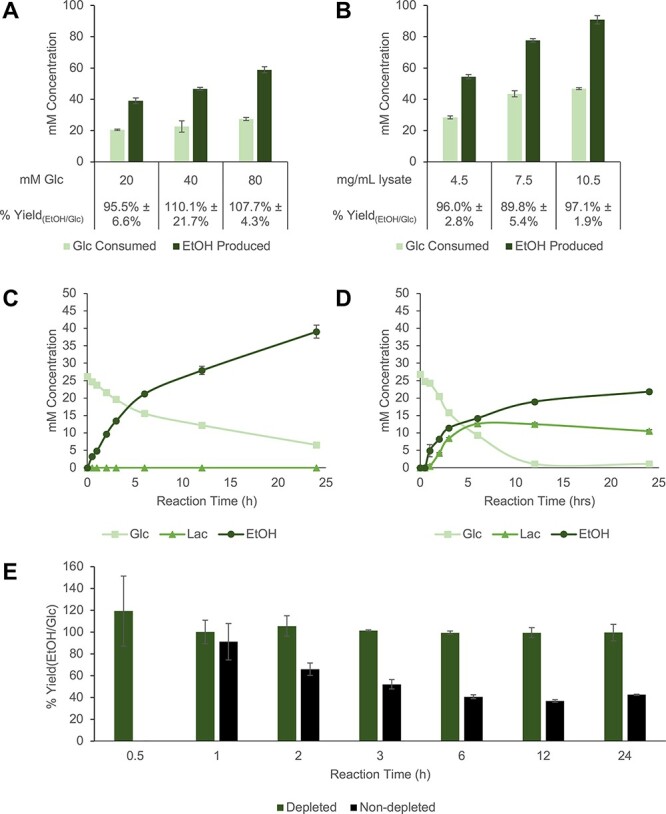
Evaluation of maximal glucose-to-ethanol conversion efficiency in the optimized system. Normalized metabolite concentrations and calculated % ethanol yields in the optimized reaction system prepared with (**A**) 4.5 mg/ml lysate and different concentrations of glucose feed or (**B**) 50 mM glucose and varying concentrations of lysate. Normalized metabolite concentrations in time-course reactions set up with (**C**) the system optimized via the block—push—pull approach and (**D**) its ND lysate counterpart (i.e. a system prepared with the optimized reaction and cultivation conditions but without removing competing reactions). These reactions were fed 25 mM glucose. (**E**) Percent ethanol yields in the optimized system and its ND lysate counterpart through time. All reactions were incubated at 37°C. Except for the time-course experiment, reactions were run for 20 h. Reactions for all conditions were prepared using depletion replicates, and error bars represent standard errors (*n* = 3).

The cell-free, block—push—pull approach enabled a 10-fold improvement in the optimized system’s ethanol yield from glucose compared to the unmodified extract (Figure 6). The consistency of the optimized system to perform at >90% yield was then assessed. First, it was determined whether flux is still driven to ethanol without high concentrations of remaining substrate. In reaction optimization experiments, only a fraction of the glucose feed (∼25 mM out of 80 mM) is consumed after 20 h (Figure 5). By Le Chatelier’s principle, the high remaining concentration of glucose could be favoring the overall forward reaction to ethanol, preventing bottlenecks and buildup of intermediates. However, optimized reactions fed with increasing concentrations of glucose convert a common concentration, 20–25 mM, of glucose to ethanol at near 100% yields (95.5% ± 6.6% with 20 mM initial Glc; 110.1% ± 21.7% with 40 mM initial Glc and 107.7% ± 4.3% with 80 mM initial Glc) (Figure 7A). Reactions are thus driven forward toward ethanol synthesis, even without excess glucose in the system, suggesting that glucose consumption and therefore ethanol concentrations are limited by another component of the reaction system. The total concentration of relevant pathway enzymes was hypothesized to limit the reaction. To this end, reactions made with increasing concentrations of blocked lysate were fed 50 mM glucose and incubated overnight. Compared to the initial reaction condition containing 4.5 mg/ml lysate, reactions with more concentrated extracts could consume more glucose and produce more ethanol while maintaining near 100% yields (96.0% ± 2.8% with 4.5 mg/ml lysate; 89.8% ± 5.4% with 7.5 mg/ml lysate and 97.1% ± 1.9% with 10.5 mg/ml lysate). (Figure 7B). Thus, increasing the concentration of extract in the mixture can be a useful strategy for improving ethanol production and possibly even the system’s productivity without impacting the yield. However, it is also conceivable that the positive effect of concentrating extract on reaction production efficiency has an upper limit due to reaction viscosity (58, 59). Alternatively, terminal enzymes may be overexpressed heterologously to help drive flux through the insulated pathway (11, 27).

To confirm that these highly efficient conversions were consistent over time, a time-course experiment was set up with 4.5 mg/ml lysate in reactions that were fed 25 mM glucose, the maximum amount of substrate this system would consume (Figure 7A). Glucose consumption inversely correlated with ethanol production, resulting in 100% ethanol yield in the blocked lysate at every time point (Figure 7C and E). These results strongly suggest that as glucose is consumed, its carbons are fully shuttled and incorporated into ethanol in the optimized system. Additionally, no significant production or consumption of acetate was observed through time (Figure S5). Furthermore, reactions prepared under the same reaction and cultivation conditions, but with ND extract, accumulated half as much ethanol and only transformed glucose to ethanol at less than half of the theoretical yield in 24 h (Figure 7D and E). These data reinforce that removing unwanted reactions was indeed necessary for effectively rewiring flux. However, this still had to be complemented by strategies to drive flux through bottlenecks and toward ethanol to realize the maximum yield. This is implied by the ∼50% yield obtained in the ND system under optimized cultivation and reaction conditions (Figure 7E), which is a ∼5-fold improvement from the <10% yield in the non-optimized system (Figure 6).

It should be noted that the ND lysate consumed glucose more rapidly than the blocked extract (Figure 7C and D). At early time points (1, 2 and 3 h), this seems to be due to the lactate-forming ability that helps AdhE turn over excess NADH to drive rapid glycolytic flux (Figure 7D). Indeed, >90% of glucose carbon was incorporated into lactate and ethanol at these earlier time points. Interestingly, when remaining glucose was completely consumed in the ND extract between 6 and 12 h, less glucose carbon was incorporated into ethanol, lactate or other fermentative products that are detectable by the HPLC method used here (Figure 7D). Whether this is because flux is being directed to non-fermentative end-products or because bottlenecks are causing the buildup of intermediates requires further investigation. Regardless, this finding highly contrasts the continuous production of ethanol from glucose with > 90% theoretical yield beyond 12 h in blocked extracts, further supporting that the workflow efficiently directs carbon flux from glucose to ethanol (Figure 7E).

## 4. Conclusions

Here, a lysate-based cell-free system was optimized to synthesize ethanol from consumed glucose with an industrially relevant % yield (>90%) (7). To our knowledge, this is the first report of metabolic flux rewiring in lysates that maximizes the yield of a targeted compound. To accomplish this, a block—push—pull approach was designed and implemented. Specifically, an efficient strategy for depleting flux-competing reactions from the lysate itself was first employed. CFME methods that could drive flux through the insulated target pathway were then applied to improve the system’s conversion of substrate to product. These strategies take advantage of the manipulable cell-free environment. That is, the lysate proteome could be curated without introducing metabolic burden from protein overexpression or deleterious mutations, and pathway activity could be rapidly modulated by directly manipulating reaction conditions. The engineering workflow here is therefore unconstrained by cell viability and enables shorter build steps in DBTL cycles. As a result, the yield of the target endogenous metabolite could be quickly maximized in a lysate, which initially synthesized ethanol from glucose at only 10% of the theoretical yield. A source strain that already had the native or introduced capacity to synthesize the targeted product with high-performance metrics was therefore not needed (27, 60, 61). This implies that time-consuming strain optimizations or the availability/discovery of already optimized strains to rewire endogenous metabolic flux is not required for engineering lysates that result in high conversion yields. This can be advantageous for reducing the number of heterologous genes introduced into the source strain, reducing manipulations to only those that are necessary for completing cell-free pathway designs. Elements of the current workflow can be feasibly applied at the bench scale to study cellular metabolism, test unconventional phenotypes or prototype potential high-yield pathway designs for *in vivo* production. It is important to note that the depletion strategy is reliable for such applications as it does not cause significant variability. However, the current method could benefit from testing other tag–resin combinations with higher specificity and an assessment of the number of enzymes that can be feasibly depleted by these approaches.

Efficient bioconversion is essential to realizing lucrative lysate-based biomanufacturing. According to estimations provided by Rasor *et al*., the synthesis of certain high-value molecules (e.g. limonene and 3-hydroxybutyrate) can be profitable in cell extracts, provided that available reaction cost–reduction strategies are integrated, and maximum conversion yields can be engineered in lysate pathways (62). The latter is demonstrated by the optimizations described here, which were designed specifically to propel flux through glycolysis for ethanol production. Appropriately designed cell-free block—push—pull approaches should similarly be able to direct carbon flow to other target metabolites. CFME methods that enrich and activate enzymes of the target pathway, integrated into the described approach as push and pull strategies, have already been applied to increase titers and productivities of lysates that synthesize a broad range of compounds (4, 12, 15, 24, 27, 61, 63). If complemented with an efficient strategy for removing unwanted reactions, it is conceivable that high yields of these metabolites can also be achieved. However, refinements should be made to the described depletion approach to enable sustainable production in extracts due to the high cost of the metal chelate affinity-based strategy (Supplemental Note). Other protein tag technologies could be considered. These tags must be small enough so that (i) their sequences can be easily inserted into genomes and (ii) they do not affect the endogenous enzyme’s physicochemical properties. Cellulose-binding domains with sequences as short as 27 amino acids long (64) can be utilized as protein tags and tested with inexpensive cellulose beads (65, 66). We estimate that lysate depletion with cellulose beads can enable profitable lysate-based biomanufacturing in the future (Supplemental Note). The *mf-*ssrA tag is also 27 amino acids in length and is sensitive to proteolysis by an exogenous *mf-*Lon protease (67). This system was previously recommended for removing flux-competing reactions post-lysis (29), but the cost of large-scale protease purification should be analyzed. An alternative strategy is the use of a native protease in *E. coli* that only degrades tagged enzymes upon cell lysis (68). An OmpT protease cleavage site (a dibasic amino acid sequence) was engineered into a strain’s endogenous cytosolic protein and cleaved by the native OmpT protease, which is localized in the outer membrane, only after membrane disruption. This method had no impact on cell growth and could thus be adapted as a block strategy for pathway yield optimization. However, this demonstration was focused on degrading a single protein for enhanced non-canonical amino acid incorporation in cell-free protein synthesis reactions (68). Using this approach to selectively lyse multiple enzymes for metabolic flux rewiring may introduce new challenges, like OmpT’s potential proteolysis of metabolic enzymes with dibasic cleavage sites as OmpT reportedly has more degradation targets in extracts (69–71). Non-native proteases like *mf*-Lon may be assessed for this type of approach, but tuning the heterologous expression of the outer membrane-localized protease would likely require strain optimization (72, 73).

This work described here illustrates the feasibility of engineering high-yield bioconversion in complex lysate environments by optimizing and combining CFME approaches. Looking forward, we anticipate that the design and execution of pathway-specific, cell-free block—push—pull workflows can achieve industrially relevant yield metrics for high-value molecules in extracts. Variations of the described workflow can be leveraged to study cellular metabolism or to prototype pathway designs before construction *in vivo*. Innovative, low-cost strategies for selective depletion of enzymes will advance commercially viable lysate-based biomanufacturing systems, providing an alternative engineering platform for rapid and sustainable bioconversion.

## Supplementary Material

ysad007_Supp

## Data Availability

The data underlying this article will be shared on reasonable request to the corresponding author.

## Material availability statement

Unique materials presented in this manuscript are available upon reasonable request and through a materials transfer agreement.
