# Supplementary material for: Rewiring cell-free metabolic flux in E. coli lysates using a block—push—pull approach
Source: Synth Biol (Oxf). 2023 Apr 17;8(1):ysad007. doi: 10.1093/synbio/ysad007 (PMC10615139; doi:10.1093/synbio/ysad007)
Supplement: ysad007_Supp [file ysad007_supp.zip › suppl_data/DinglasanDoktycz-CellFreeYield-OxfordSynBio-SupplementaryFigsPlusNote-Final-040423.pdf]

Online Supplement

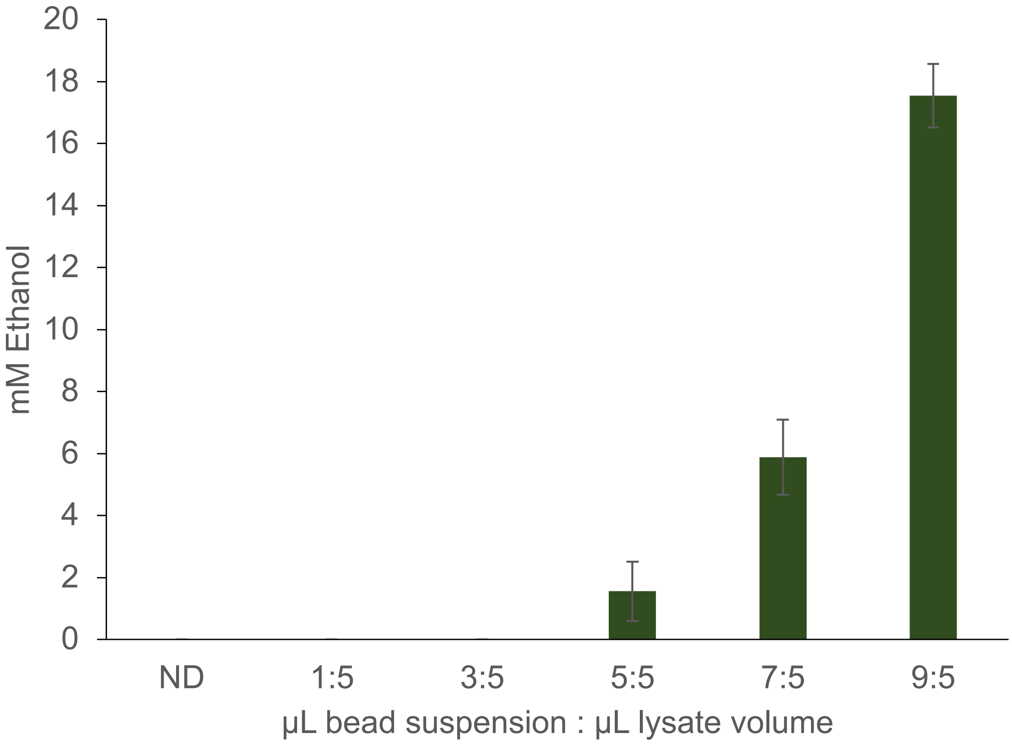

Figure S1. Residual ethanol from resin suspension storage buffer carried over to time zero lysate reaction set-ups.

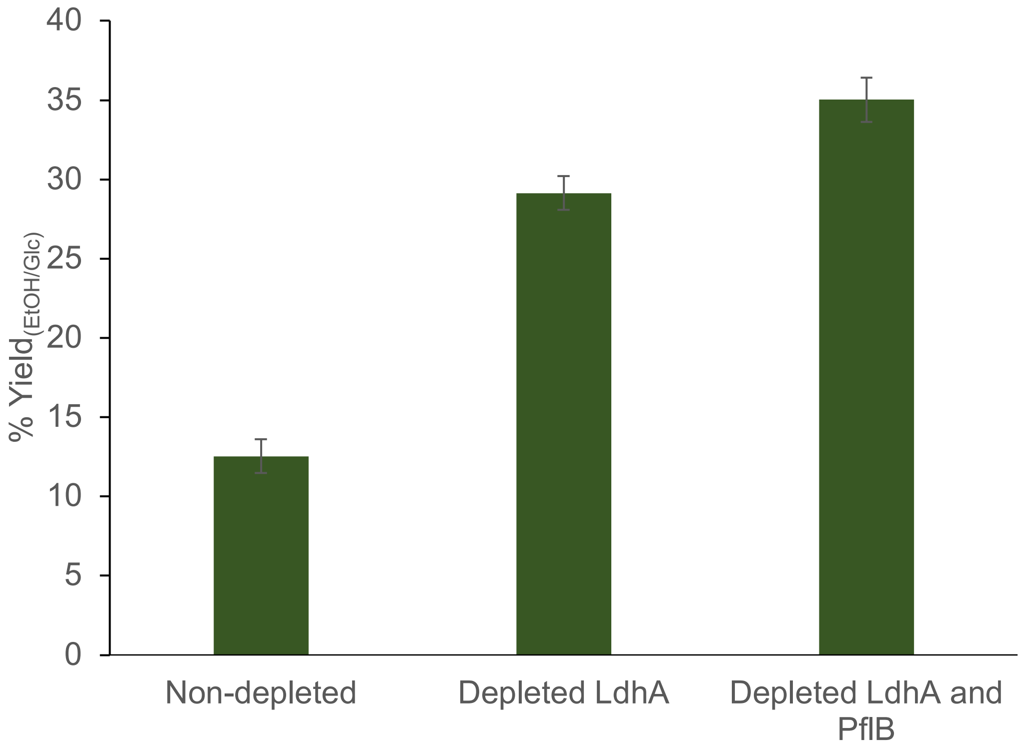

Figure S2. Contribution of PflB depletion to the improvement of ethanol yield in LdhA depleted lysates. Lysates were derived from a strain that endogenously expresses both 6xHis-tagged LdhA and PflB or 6xHis-tagged LdhA alone. Lysates were depleted by mixing 200 µL lysate with washed Co^{2+} beads from a 200 µL resin suspension. Reactions were initiated with 100 mM glucose and incubated for ~ 20 hrs at 37˚C along with non-depleted control reactions. Depletion reactions were used for all conditions and error bars represent standard error of the mean (n=3).

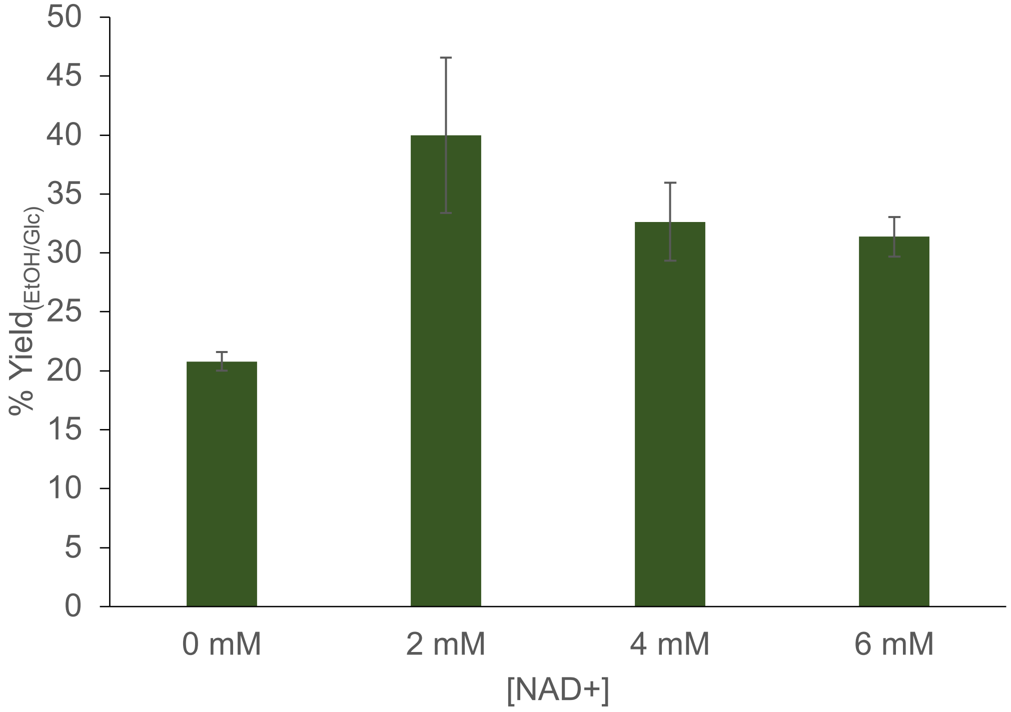

Figure S3. Additional NAD^+ was supplemented to reactions prepared with blocked lysate, 100 mM Glucose, 0 mM CoA and ATP, but had no improvement or significant effect on % ethanol yield from glucose beyond 2 mM NAD^+. Reactions were incubated at 37˚C for 20 hrs. Depletion replicates were used for all conditions and error bars represent standard errors (n=3).

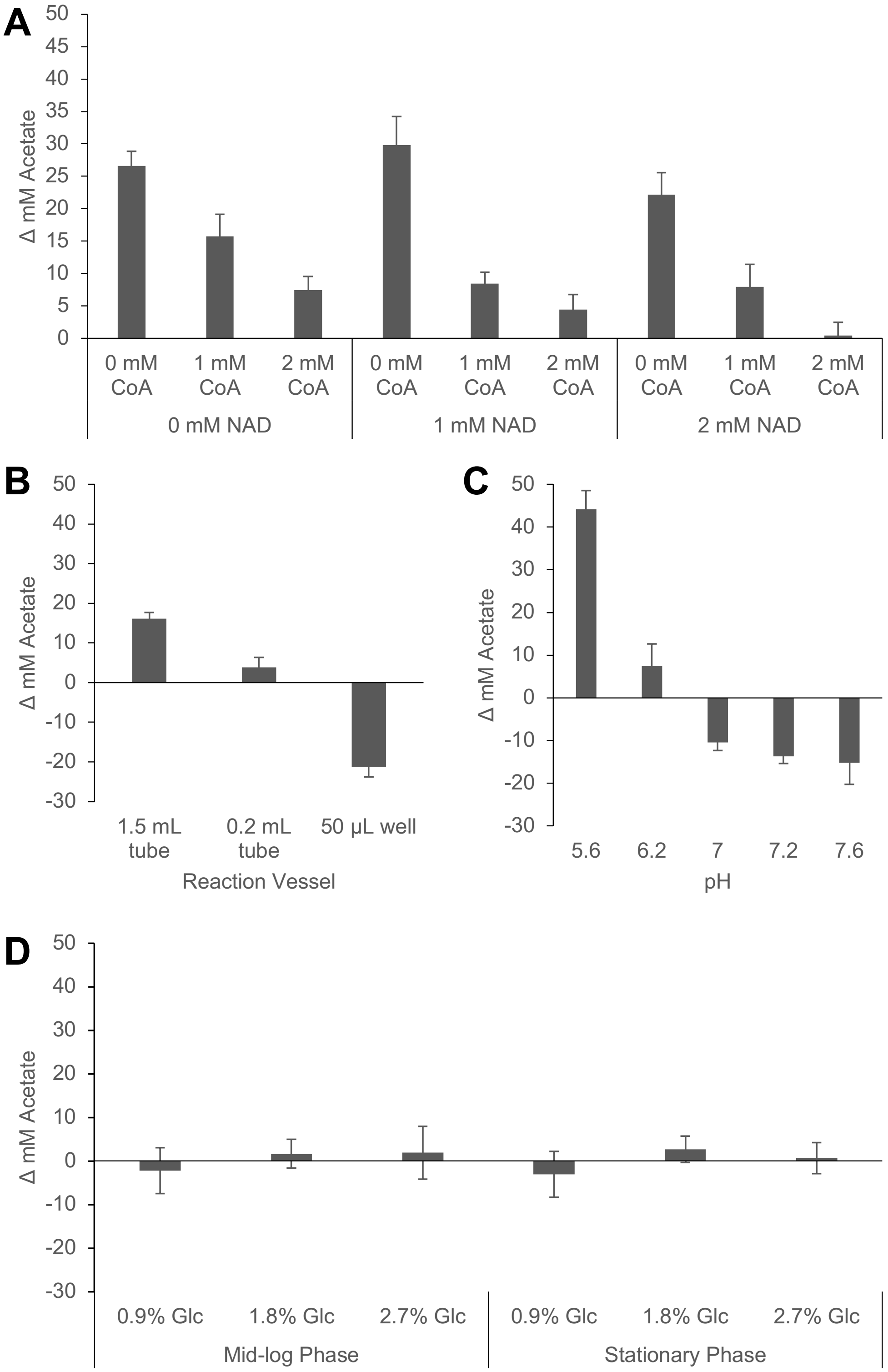

Figure S4. Acetate production or consumption in reactions that were **A)** initiated with 0 mM ATP and various combinations of NAD^+ and CoA, **B)** incubated in different enclosed reaction vessels, **C)** initiated with varying pH levels, **D)** prepared with lysates derived from strains grown in different cultivation conditions. Depletion replicates were used for all conditions and error bars represent standard errors (n=3).

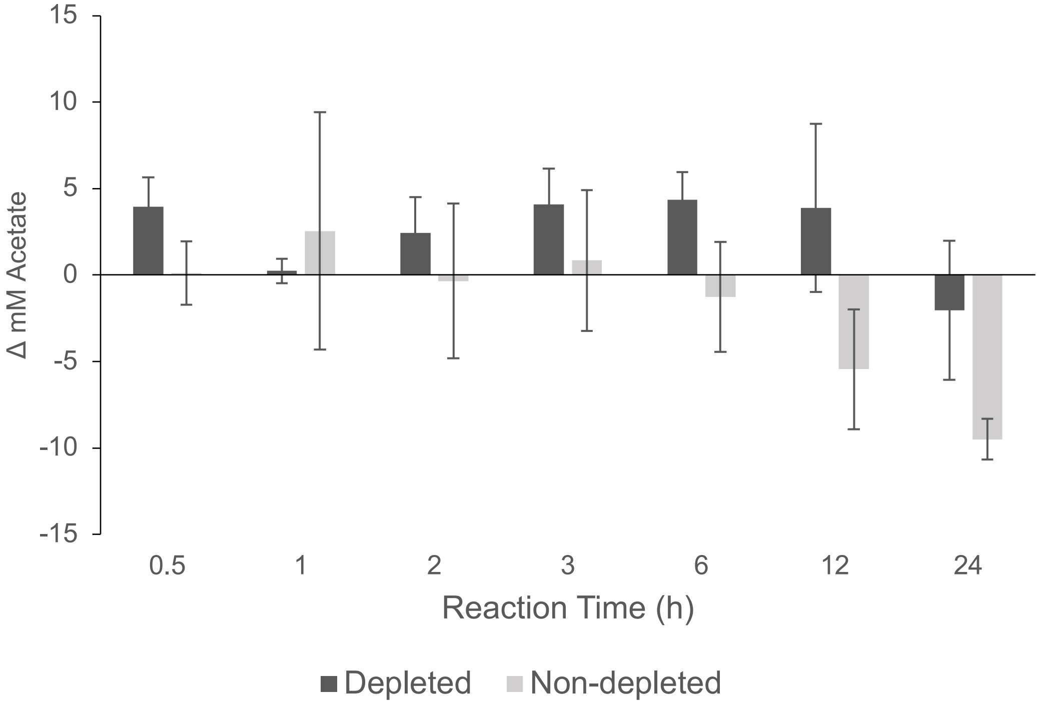

Figure S5. Acetate production or consumption in the optimized system and its non-depleted lysate counterpart, calculated as the difference between acetate concentrations at a particular timepoint and at time zero reactions. Depletion replicates were used for all conditions and error bars represent standard errors (n=3).

Supplemental Note:

The cost of the current selective enzyme depletion approach (i.e., block strategy) is evaluated using reaction preparation costs that were already calculated by Rasor et al[1]. Briefly, they estimate the breakeven cost of a reaction that produces high-value targets (e.g., limonene and 3-hydroxybutyrate) to be $275.00/L reaction. They define the breakeven reaction cost as "the cost of reaction required to start having a return on investment at a given max theoretical yield." With innovative cost-reducing strategies (i.e., efficient cofactor utilization, single-carbon substrates, high-density cultivation) and neglecting labor/capital costs, the price of reaction preparation can be reduced to $90.00/L reaction in the future.

In the current approach, equal volumes of lysate and Co^{2+} resin suspension (Thermo Scientific #89964) are needed to eliminate byproduct-forming activity. The resin is $132.00/10 mL bottle. The resin itself therefore adds $0.40 to the preparation of a 30 μL reaction. Assuming $90.00/L reaction ($0.0027/30 μL reaction), the total cost for preparing a system with the modified extract is $0.4027/30 μL reaction or $13,423.00/L reaction. Potential bulk pricing and other economies of scale may reduce these costs, but the present depletion strategy is unlikely to be viable for commercialization.

Alternative pre-purification strategies may enable sustainable yield engineering efforts. Islam et al. recently reported an approach involving the purification of proteins via cellulose binding domains (CBD) and microcrystalline cellulose (Sigma-Aldrich #310697)[2]. In their method, 700 mg of microcrystalline cellulose, which costs $49/50 g, could be used to make 2000 μL bead volume. If each target endogenous enzyme is engineered to display a CBD and an equivalent volume of bead volume is needed to eliminate byproduct-forming activity, the material costs $0.0010/30 μL. Assuming $90/L reaction, the total cost for preparing a system with the modified extract is $0.0040/30 μL reaction or $133/L reaction. Even without considering economies of scale, this is well below the breakeven reaction cost for high-target chemicals. With method optimization, it is possible that less microcrystalline cellulose is necessary to inactivate byproduct-forming activity, further reducing preparation costs.

(1) Rasor, B. J., Vögeli, B., Landwehr, G. M., Bogart, J. W., Karim, A. S., and Jewett, M. C. (2021) Toward sustainable, cell-free biomanufacturing. *Curr. Opin. Biotechnol. 69*, 136–144.
(2) Islam, M. R., Kwak, J. W., Lee, J. soo, Hong, S. W., Khan, M. R. I., Lee, Y., Lee, Y., Lee, S. W., and Hwang, I. (2019) Cost-effective production of tag-less recombinant protein in Nicotiana benthamiana. *Plant Biotechnol. J. 17*, 1094.
