# Supplementary material for: Rewiring cell-free metabolic flux in E. coli lysates using a block—push—pull approach
Source: Synth Biol (Oxf). 2023 Apr 17;8(1):ysad007. doi: 10.1093/synbio/ysad007 (PMC10615139; doi:10.1093/synbio/ysad007)
Supplement: ysad007_Supp [file ysad007_supp.zip › suppl_data/DinglasanDoktycz-CellFreeYield-OxfordSynBio-TableS1-040423.docx]

**Table S1.** Number of cycles needed to wash ethanol off different resin suspension volumes with S30 buffer (for 200 µL lysate).

| **Volume resin suspension (µL)** | **Number of wash cycles** |
| --- | --- |
| 40 | 2 |
| 120 | 2 |
| 200 | 3 |
| 280 | 5 |
| 360 | 7 |
